# Traditional uses of medicinal plants used by Indigenous communities for veterinary practices at Bajaur Agency, Pakistan

**DOI:** 10.1186/s13002-018-0212-0

**Published:** 2018-01-29

**Authors:** Muhammad Abdul Aziz, Amir Hasan Khan, Muhammad Adnan, Habib Ullah

**Affiliations:** 1 Department of Botany, Kohat University of Science and Technology, Kohat, 26000 Pakistan; 2 Department of Botany, Shaheed Benazir Bhuto University Sheringal, District Dir (Upper), 18000 Pakistan; 3 Department of Zoology, Abdul Wali Khan University, Mardan, 23200 Pakistan

**Keywords:** Folk knowledge, Indigenous communities, Livestock diseases, Fic, Phytopharmacological studies

## Abstract

**Background:**

The pastoral lifestyle of Indigenous communities of Bajaur Agency is bringing them close to natural remedies for treating their domestic animals. Several studies have been conducted across the globe describing the importance of traditional knowledge in veterinary care. Therefore, this study was planned with the aim to record knowledge on ethnoveterinary practices from the remote areas and share sit with other communities through published literature.

**Methods:**

Data was gathered from community members through semi-structured interviews and analyzed through informant consensus factor (Fic) to evaluate the consent of current ethnoveterinary practices among the local people.

**Results:**

In total, 73 medicinal plants were recorded under the ethnoveterinary practices. Most widely used medicinal plants with maximum use reports (URs) were *Visnaga daucoides* Gaertn., *Foeniculum vulgare* Mill., *Solanum virginianum* L., *Withania somnifera* (L.) Dunal, *Glycyrrhiza glabra* L., and *Curcuma longa* L. New medicinal values were found with confidential level of citations for species including *Heracleum candicans* and *Glycerhiza glabra*. Family Apiaceae was the utmost family with high number (7 species) of medicinal plants. Maximum number of medicinal plants (32) was used for gastric problems. High Fic was recorded for dermatological (0.97) followed by reproductive (0.93) and gastrointestinal disorders (0.92). The main route of remedies administration was oral.

**Conclusions:**

Current study revealed that the study area has sufficient knowledge on ethnoveterinary medicinal plants. This knowledge is in the custody of nomadic grazers, herders, and aged community members. Plants with new medicinal uses need to be validated phytochemically and pharmacologically for the development of new alternative drugs for veterinary purposes.

## Background

The historical utilization of plants as health remedies both for human and animal is centuries old. It has been recognized that plants have the capacity to combat several types of diseases ethnoveterinary medicines, a term generally used for folk skills, beliefs, knowledge, practices, methods related to animals’ health, and cure of various ailments in the rural areas [1]. Ethnoveterinary practices have achieved immense significance for the last decade owing to the discovery of some effective ethnoveterinary products [2]. The utilization of traditional remedies poses a cheaper, easier, and sustainable alternative to synthetic drugs and pharmaceuticals [3]. It has been reported that due to lack of proper animal husbandry practices, about 30–35% of the losses occur in the animals’ breeding sectors especially in developing countries [4], where the rural people are heavily dependent on livestock farming for their livelihood activities [5]. The Indigenous communities living in rural and mountainous territories of developing world consider livestock a vital source for economy, social security, and food and is thought to be a symbol of prestige for a particular family [6].

Livestock being as a subsector contributes around 56% of value addition in the agriculture sector and approximately 11% towards the gross domestic product (GDP). About 30 million people living in the rural areas of the country are involved with the livestock subsector [7]. Hence, livestock raring plays a significant role in poverty reduction strategies. According to the report of economic survey of Pakistan [8], the national herd of Pakistan includes 53.8 million goats, 29.6 million cattle, 27.3 million buffalos, 26.5 million sheep, and 0.9 million camels. People residing in the remote areas utilize medicinal plants for livestock’s health. Particularly, the conventional lifestyle of nomadic and pastoralists makes it difficult for them to reach veterinary extension services due to high costs and less availability of allopathic medicines [9].

In South Asia, several ethnoveterinary studies have been conducted [10–18] including Pakistan [6, 9, 19–26]. However, scarce studies on ethnoveterinary medicines have been reported from the Federally Administrated Tribal Areas (FATA) of the country. The tribal areas mainly comprised of mountainous territories where people use medicinal plants to treat livestock’s diseases. Traditional ethnoveterinary knowledge is mainly transmitted orally from one generation to another generation in the form of folk remedies, drawing stories, poems, drawing stories, folk myths, songs, and proverbs. This transmission of Indigenous knowledge through oral way faces critical threats of extinction. Therefore, it is necessary to record, document, and encourage the ethnoveterinary medication and integrate them into the existing animal health care services [5].

Bajaur agency is among one of the Federally Administrated Tribal Areas (FATA) of Pakistan having diversity of medicinal plants being used for the livestock’s healthcare services. Due to remote nature and lack of quality education, the area has been little explored for the scientific documentations of traditional knowledge. There is a dire need to explore the folk knowledge about the utilization of herbal remedies for veterinary practices prior to being extinct. Hence, the current study was planned to investigate and document the traditional ethnoveterinary knowledge and practices and release it from the custody of knowledge bearers for sharing it with other communities through publish literature.

## Methods

### Study area

Bajaur (Khar: headquarter) is the smallest agency of the FATA having a total area of 1290 km^2^. It shares 52 km border with Afghanistan, which is of great importance to Pakistan and the region. The study area lays at an altitude of 1126 m above the sea level and geographically exists between 34°–30° and 34°–58° latitudes and 71°–11° and 71°–30° longitudes. The Agency is surrounded to the west by Kunar Valley of Afghanistan being separated by the rugged Hindukush hills and other mountain passes known as Nawa Pass, Ghakhi Pass, and Letai Sar being the notable ones. The agency borders on south with Mohmand agency, on east with Lower Dir District and the Panjkora River, and on north with the watershed between Bajaur Agency and District Dir. Moreover, the agency is situated at the extreme end of the Himalayan Range. The areas dominated by agricultural lands are receiving about 800 mm of average rain fall per annum. The two main tribes of Bajaur Agency known as Tarkani and Utman Khel are mainly populated into seven Tehsils including Barang, Nawagai, Khar, Mamund, Salarzai, Utman Khel, and Chamarkand. By profession, mostly, the people are farmers, teacher, drivers, and doing small scale businesses and jobs inside/outside the country. Almost every household has a herd of domestic animals for socioeconomic gains. There are only three degree-level colleges and five higher secondary schools. Moreover, there are only two government hospitals in the study area, while most people are deprived of modern health facilities, which justify their reliance on local herbalists (Hakims). The study area consists of one veterinary hospital and 20 small dispensaries to treat the domestic cattle. However, the local people still rely on traditional recipes due to larger distances from the aforementioned health centers. The dominant vegetation in the area is comprised of *Ailanthus altissimo*, *Eucalyptus camaldulensis*, *Ficus carica*, *Melia azedarach*, *Morus indica*, *Morus nigra*, *Olea ferruginea*, *Pinus roxburghii*, *Quercus baloot*, and *Rumex hastatus*.

### Ethnomedicinal data collection and ethnographic composition

In the month of April, respondents were targeted based on their strong reputation in the field of ethnomedicinal knowledge while field survey was conducted from May to August 2016. Field visits were carried out prior to medicinal data collection in order to acknowledge the cooperation of the Indigenous communities. Mr. Amir Hasan Khan, the local occupant of the area, visited different sites with his volunteer team including a taxonomist and a pharmacist. He arranged several meetings with the local representatives known as Maliks, to whom objectives of the study were presented.

A semistructured questionnaire was developed to gather knowledge on ethnoveterinary plants by following the method adopted by Martin [27]. Mostly, the folk knowledge was gathered from nomads, farmers, and aged community members. The interviews were conducted at various places and in the local language called “Pashto.” Each informant was acknowledged by presenting the main theme of the study to them in order to gain their consent and trust, which allowed the informants to talk more freely and openly. The recorded information was once again redisplayed to the informants to avoid errors and falsification.

**Fig. 1:**
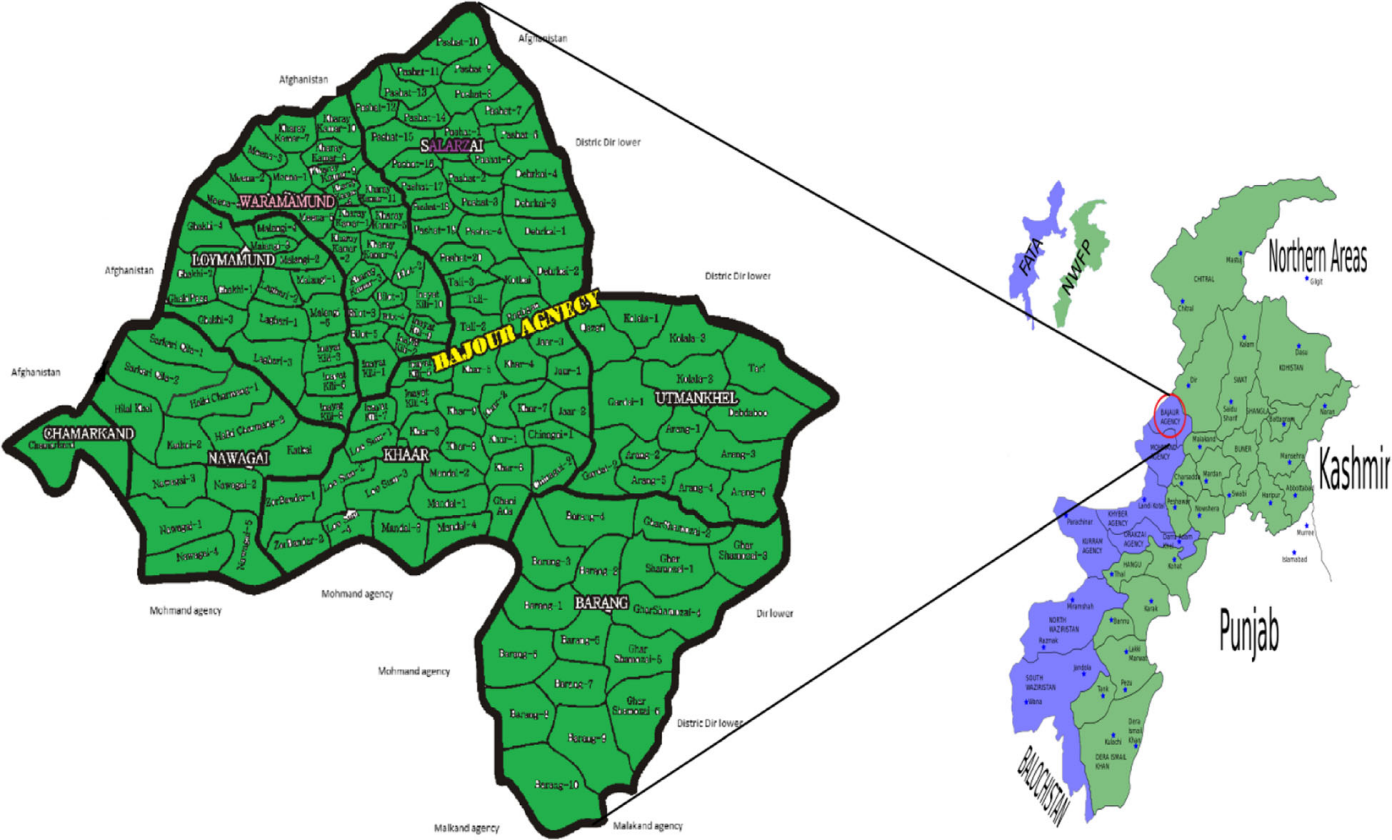
Map of the study area and area location in Pakistan

Data was collected from different sites known as Pashat, Tali, Inayat Kali, Ghar Shamozai, Loe Sum, Barang, Mandal, Khar, Mamund, and Salarzai. Accordingly, the sites were categorized into foot hill villages and mountainous villages (Fig. 1). A total of 80 key respondents were selected belonging to different age groups, i.e., 68 males and 12 females (Table 1). The selection of respondent was based on their high reputation with respect to traditional knowledge on ethnoveterinary plants. Continuous relationships were maintained with the Indigenous communities throughout the course of survey for the strong validation of traditional knowledge.

**Table 1:** Indicating the number and details of informants

Category	Total	%
Gender		
Male	68	85
Female	12	15
Age groups		
35–50	27	34
> 50	53	66
Occupation		
House wives	12	15
Herders	40	50
Farmers	28	35

### Preservation and taxonomical verifications of plant species

Surveyed ethnoveterinary medicinal plants were collected and identified by taxonomist at the Department of Botany, Shaheed Benazir Bhuto University Sheringal, District Dir (Upper), Khyber Pakhtunkhwa, Pakistan. Species botanical names and their family names were corrected and verified through the website www.kew.org/mpns. After collection, plants were pressed and dried under the shade, were poisoned (1% HgCl_2_ solution), and were mounted properly on the herbarium sheets for future reference. Each herbarium sheet was labeled with a voucher number and submitted to the aforementioned department [28, 29].

### Data analysis

For each of the specie, use reports (URs) (citations) were counted. UR may be defined as the utilization of part of a plant species for a particular disease mentioned by an informant. To determine the informant consensus factor (Fic), the reported species were arranged in various groups according to the ailment treated [11]. Ten ailment categories were prepared from the data. To calculate the Fic, we used the formula, i.e., Fic = Nur − Nt/Nur − 1. Here, Nur indicates the number of citations in each use category and Nt represents the number of species cited.

## Results and discussion

### Prospects and challenges to traditional ethnoveterinary knowledge

Indigenous communities play significant role in reporting traditional uses of medicinal flora. Indigenous knowledge can be used as a tool to conserve and maintain the green diversity, and could be further utilized for scientific validation [12]. During the 32nd session of United Nations Educational, Scientific and Cultural Organization (UNESCO), traditional knowledge on ethnoveterinary medicines was declared an important part of cultural heritage, which is required to be brought under study, sustenance, and protection [30].

Indigenous communities at Bajaur Agency are dependent on livestock for supporting their livelihood. Medicinal plants have a pivot role in the treatment of livestock’s ailments in the area. Usually, this treatment process depends either on the traditional knowledge being orally transmitted to the current generation of local people from their ancestors or through personal experiences. Previous scientific literature has focused on the correlation of traditional medical expertise to ethnobotanical knowledge for the treatment of human ailments [31, 32], although the same plants may be used to treat livestock [33, 34]. In our study, we have observed that the herders, farmers, and older community members are more equipped with traditional knowledge and familiar with veterinary medications, diagnosis process, and treatment.

**Fig. 2:**
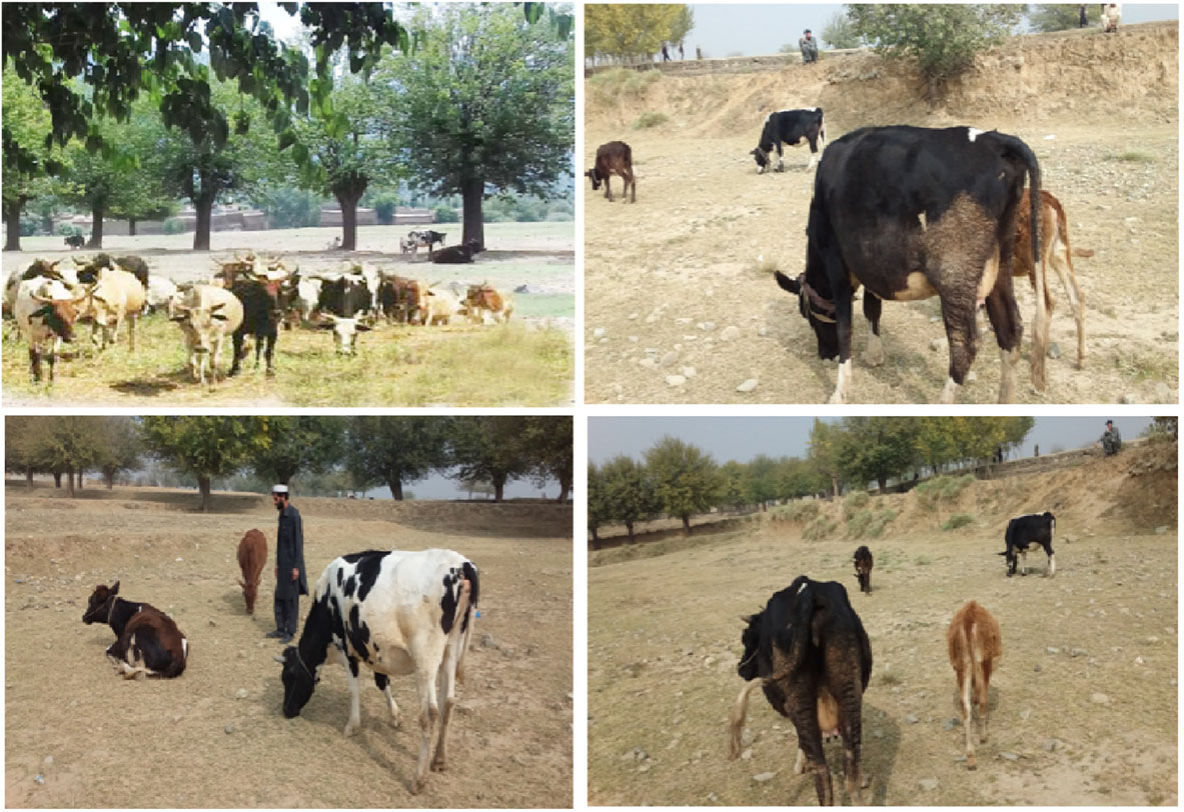
Images of some of the animals treated with medicinal plants

**Fig. 3:**
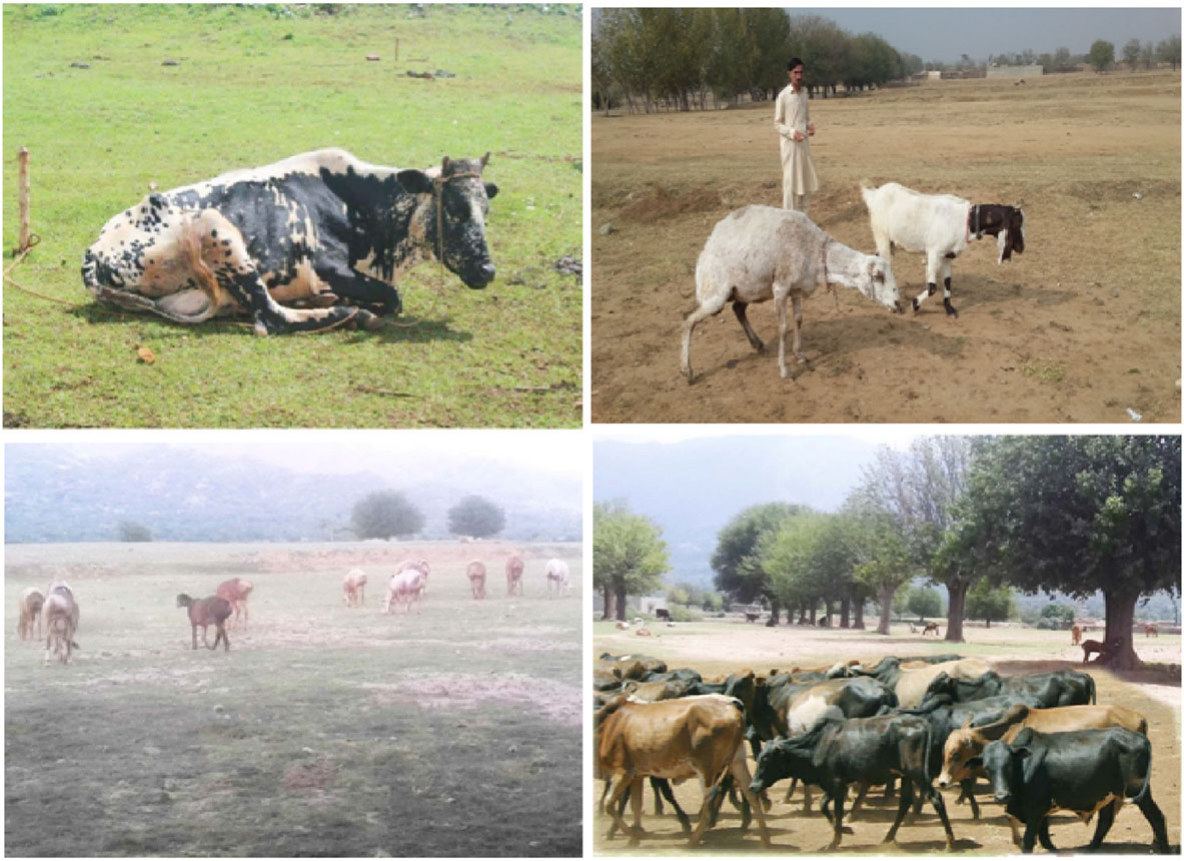
Images of some of the animals treated with medicinal plants

Indigenous people of the study area are rich in traditional knowledge on veterinary medicines, which may be due to their close observation on domestic animals being considered as an important part of traditional lifestyle. Most commonly, the male community member grazes herds of animal, while females take part in households’ management. Figures 2 and 3 showed some of the images of the grazed domestic animals, which are treated with medicinal plant in the area. Other studies have explained this in a different way that men due to close proximity tend to know more about the animal behavior than women [31].

People of the study area use plants not only for medicinal purposes to treat their domestic animals but also as a fodder. Local community also prevents their animals from such nutrition, which is not healthy in certain conditions and seasons. One may consider this prevention to be a part of ethnoveterinary practices. Nutrition is playing an important role in ethnoveterinary practices in both prevention and cure of domestic animals [35]. Livestock usually ingests some extra and non-important food substances in the green fodder, which could be termed as food medicines or medicinal food [36]. Studies have highlighted the importance of “food as medicines” in the context of local traditional knowledge; however, possible health advantages of food in ethnoveterinary methods need further attention [37]. Testing the nutritional status of each traditional ethnoveterinary remedy is not necessary; however, it is essential to evaluate the biological efficacy from the phytochemical, pharmacological, toxicological, and clinical perspectives for wider application. A considerable proportion of the documented uses of plant taxa in our study are in accordance with the established pharmacological effects [36].

The prevailing Indigenous ethnoveterinary knowledge in the study area is facing certain constrains leading it towards extinction. As an example, the nature of traditional knowledge is making it more difficult to learn and then transfer it in an accurate way. Furthermore, practicing traditional therapies are not being respected by the new generation. Other challenges include low literacy rate in the study area, no proper documentation of Indigenous knowledge, and introduction of modern allopathic medicines, rapid technological advancement, and environmental degradation. Similar kinds of threats have also been reported in other communities across the world [38–40]. Informants with little education were found less familiar to the traditional knowledge while people having no formal education were more responsive in this regard. Some studies have found that education can be correlated with expertise either positively [41, 42] or negatively [43], while others found no relationship [44]. Moreover, it is also ambiguous to determine the effect of “modernity” on the loss of ethnomedicinal knowledge. Modernity has an established association with greater medicinal competence in Dominica [45] but appeared unrelated to variation in expertise among Tsimane horticulturalists in Bolivia [41]. Furthermore, it is also unclear whether correlation of expertise exists between ethnomedicinal knowledge and ethnoveterinary approaches; however, livestock keepers hold extensive knowledge related to disease prevention, diagnosis, and both traditional and novel biomedical treatments [26]. In summary, despite maintaining knowledge on ethnoveterinary practices by the locals, the tendency to utilize modern pharmaceuticals is increasing day by day. Hence, the conservation of ethnomedicinal knowledge by the local communities is extremely important for the livestock’s health in the remote areas.

### Ethnoveterinary medications and their cross-cultural analysis with nearby regions

The use of plants for medical purpose to treat a wide array of maladies emanates traces since the recorded history and even before. In our study, 73 plant species belonging to 43 families were documented. Table 2 presents details on the documented medicinal plants including their botanical names, vernacular names, family names, specimen numbers, parts used, medicinal uses, and use reports. Family Apiaceae (7 species) has the high number of individual species used in ethnoveterinary practices followed by Fabaceae (5 species). Other studies have also reported Apiaceae as the dominant plant family being used in traditional medications [37, 46]. The rationale of high use of Apiaceae species in the current study, though based on traditional evidence, may be referred to their chemical constituents such as phenolics, poly phenolics, lectins, alkaloids, terpenoids, and essential oils, which carry antimicrobial potential [47]. Due to the predominance of sheep, goats, cows, and donkeys in the study area, we have specifically recorded the ethnoveterinary practices used for the treatment of these four types of domestic animals. Key informants declared extensive uses of *Visnaga daucoides* Gaertn. (49 URs), followed by *Foeniculum vulgare* Mill. (47 URs), *Solanum virginianum* L. (43 URs), *Withania somnifera* (L.) Dunal (38 URs), *Glycyrrhiza glabra* L. (33 URs), and *Curcuma longa* L. (29 URs) (Table 2). Medicinal plants with high URs strengthen the concept that such species are more significant to the local population and useful in sharing the traditional knowledge with one another in the area. In our study, *V. daucoides* is used to treat diarrhea, abdominal pain, and retained placenta in domestic animals. The usage mode of ethnoveterinary plant species by one ethnic community is different from other communities due to difference in traditional knowledge [10, 15]. Previous literature has shown that decoction of the fruit of *V. daucoides* is used during abdominal pain, which is used to enhance body temperature in the study area [21, 48]. In the same way, the *F. vulgare* is considered as a strong appetite and sedative. In other cultures across the globe, *F. vulgare* is used for various livestock problems. For instance, this plant is effective in digestion and diarrhea, when mixed with *Camellia sinensis*, *Trachyspermum ammi*, ghee, and sugar [6, 21]. Pneumonia is also being treated by giving its seeds to the animals [24], while other uses include galactagogue and ruminative [49]. Various parts of *S. virginianum* are taken for the treatment of cough, fever, milk production, and pain. There is scarce literature on the use of *S. virginianum* as galactagogue, which shows the unique use of this plant species in the study area and familiarity of local population through longtime experiences. Published literature has indicated that the plant is also used for wound healing process [20] fever, cough, and intestinal infections [20]. Roots and leaves of *W. somnifera* are given to sheep, cow, and buffalo for milk production and used as antipyretic and sexual tonic. Indigenous populations comprising of various cultures residing in Lesser Himalayas (Pakistan) use *W. somnifera* for bovine mastitis [6], while in Ethiopia, this plant is being used to protect animals from bad evils [50]. The plant has carminative effects and is used to remove the flatulence [25]. Additionally, this plant is used as refrigerant and for abdominal pain, digestion, jaundice, skeleton-muscular ailments, and wound healing against sunstroke [26]; for treating diarrhea [22]; for trypanosomiasis [9]; and for anorexia [50]. Informant reported *G. glabra* as galactagogue and enhances the rate of fertility. Mussarat et al. [22] reported that this plant is culturally used for the treatment of cough by the Indigenous communities residing near the Indus River, Pakistan. However, from the literature, no conclusive evidence was found on the reported uses of *G. glabra* in our study. Such evidence-based observations could justify the idea of cultural diversity across the regional level in plant remedies. Previous studies related to the human’s uses of *G. glabra* have demonstrates its effectiveness in the treatment of sex hormone imbalances and menopausal symptoms in women [51]. In the current investigation, rhizome of *C. longa* is used as antiparasitic and treating genital infection and problems. In other cultures, across the country, the dried rhizome of *C. longa* is mixed with eggs and given for mastitis [22], jaundice, and skeleton muscular ailments [26]. Decoction of its leaves is mixed sugar, which is used as wound healing agent [6, 19]. A root of *C. longa* is used for hoof problems and sore joints [52]. In our study, the mustard oil is mixed with whey and is taken orally to relieve abdominal pain. The cultural ethnoveterinary uses from the Lesser Himalayas (Pakistan) include that the oil extracted from *B. rapa* seeds is utilized for stomach disorders, eye infection, and skin diseases [6]. Furthermore, *Brassica rapa* L. seeds are used for the retention of fetal membrane, while its oil is effective in treating genital prolepses and sores [53]. This plant is also used in placental retention and mastitis and as antiparasitic [19]; myiasis, mange, and helminthiasis [20]; and flatulence [49]. All these research-based findings showed that the same medicinal plants are being used in different parts of the country; however, their uses differ from area to area and from culture to culture [48]. The ethnoveterinary plants use by one ethnic community is almost different from other communities due to several reasons. To make a comprehensive comparative cultural diversity analysis of plant utilization in ethnoveterinary practices, we have selected a study conducted by Aziz et al. [48] in the FATA region of Pakistan. In comparison, we have found that most widely used medicinal plant species in our study are *V. daucoides*, *F. vulgare*, *S. virginianum*, *W. somnifera*, *G. glabra*, and *C. longa*. While according to Aziz et al. [48], the ethnic communities in South Waziristan Agency are widely utilizing plant species such as *B. rapa*, *Punica granatum*, *Capparis decidua*, *Mentha longifolia*, *Withania coagulans*, and *C. longa*, during comparative analysis, it was found that only 15 medicinal plants were commonly used in both regions for ethnoveterinary practices, which include *Acacia modesta* Wall, *Allium cepa* L., *Allium sativum* L., *B. rapa*, *Calotropis procera* (Aiton) Dryand., *Cannabis sativa* L., *Chenopodium album* L. *C. longa*, *F. vulgare*, *Juglans regia* L., *Nicotiana tabacum* L., *Peganum harmala* L., *Quercus oblongata* D. Don, *Trachyspermum ammi* (L.) Sprague, and *V. daucoides*. Certain variations in the utilization of these plants and their parts were observed in both areas. For instance, the bulb of *A. cepa* is used as galactagogue by Waziristanian communities while in Bajaur, it is used to treat digestive problems. *A. sativum* is utilized for genital prolapsed while the same plant is used as sexual tonic for animals in Bajaur Agency. The seeds of *B. rapa* are widely used as appetizer and tonic and for cough, seasonal allergies, stomach disorders, and skin infections in South Waziristan Agency, while in the other region, it is used only against gastro-intestinal disorders. The Indigenous communities at South Waziristan Agency consider the leaves of *C. procera* useful in joint pain while on the other side, the residents of Bajaur Agency used the latex against skin problems. *C. album* is used for wound healing and flatulence at Waziristan while as stomachic at Bajaur Agency. *J. regia* is given for the retention of placenta at Waziristan while gastric problems in Bajaur. *P. harmala* is extensively used for gastrointestinal problems, as antiparasitic, and for skin diseases by Waziristanian communities, while it is used only for the riddance of external parasites in Bajaur. The possible reason for low consensus of the two regions in ethnoveterinary medicinal plants may be due to unique vegetation and distinct socio-cultural values.

**Table 2:** Medicinal uses of local flora for ethnoveterinary uses

Family	Botanical name “voucher no.”	Plant local name	Parts used	URs	Medicinal value
Amaranthaceae	*Beta vulgaris* L. “SBBU-33”	Choqandar	Whole plant	6	A whole plant is subjected to powder and is combined with flour and black tea to treat digestive problems especially in cow and buffalo.
Amaryllidaceae	*Allium cepa* L. “SBBU-32”	Piaz	Bulb	14	Bulb of the herb is crushed and added milk for orally given to animals (6–8 days) for curing digestive complaints.
	*Allium sativum* L. “SBBU-16”	Oga	Bulb	13	Bulb is crushed and mixed with way to administered orally for several days in order to rate of fertility in domestic animals.
	*Narcissus tazetta* L. “SBBU-59”	Gul-E-Nargas	Leaves	10	Along with gurr and flour, fresh leaves (¼ kg) are boiled and orally given to livestock for the retained placental removal.
Apiaceae	*Carum carvi* L. “SBBU-24”	Zeera	Fruit	14	Tea is prepared from its fruit (¼ kg), and then, it is combined with flour and given to cattle for 3 days in order to treat gastric problems.
	*Cuminum cyminum* L. “SBBU-63”	Zankai	Fruit	12	Half kilogram of fruit is boiled in black tea and orally given for 15–20 days on daily basis for the expulsion of intestinal worms and treated gastric problems.
	*Eryngium biehersteinianum* (M. Bieb.) Nevski “SBBU-5”	Yakandaz	Stem and leaves and stem	23	Powder of its stem and leaves is orally given for the treatment of liver problems up to the duration of 8–12 days.
	*Foeniculum vulgare* Mill. “SBBU-61”	Kagelani	Fruit, leaves	47	Decoction is made from it fresh leaves and fruit (150–200 g), and then, it is combined with gurr, given orally to livestock for appetite and as sedative for the duration of 5 to 6 days.
	*Heracleum candicans* Wall. ex DC. “SBBU-17”	Skhwara	Roots	13	Fresh root of the plant (200 g) is combined with wheat flour and made to paste which is orally given to goat, cow, and sheep as sexual tonic and to enhance the rate of fertility up to 3 days.
	*Trachyspermum ammi* (L.) Sprague “SBBU-60”	Ajwain	Fruit	19	Seeds (¼ kg) of the plant, *Allium cepa*, wheat flour, and *Foeniculum vulgare* are thoroughly mixed. The resultant blend is then orally given (15 days) and is considered as good appetizer.
	*Visnaga daucoides* Gaertn. “SBBU-48”	Sparkai	Fruit	49	Tea is made from its fruit and given orally to sheep, goat, cow, and buffalo while treating diarrhea, abdominal pain, and retained placenta. The remedy is constantly utilized for the duration of 3 days.
Apocynaceae	*Calotropis procera* (Aiton) Dryand. “SBBU-18”	Spalmai	Leaves	21	Plants’ fresh leaves are taken and decoction is made, and after that, the decoction is combined with “*Ajuga integrifolia*” and is used for dermal parasites for 3 to 5 days.
	*Nerium oleander* L. “SBBU-22”	Gandiaray	Leaves	19	To relieve the external parasite, the decoction of its leaves is used for animal bathing especially goat and cow.
Asteraceae	*Artemisia absinthium* L. “SBBU-52”	Spara Botay	Whole plant	5	Whole is subjected to powder and mixed with gurr and wheat and is used as anthelmintic.
	*Artemisia scoparia* Waldst. & Kit. “SBBU-67”	Spara Botay	Leaves	13	Decoction is made by taking ½ kg of leaves which is mixed with gurr and administered orally daily (for 2 to 3 days) about 1 to 2 glasses for the treatment of gastric complexities, blood purification, and skin problems in goat, sheep, and cow.
	*Launaea procumbens* (Roxb.) Ramayya & Rajagopal “SBBU-44”	Shodapai	Whole plant	9	Fresh plant is fed to animals, i.e., goat, sheep, and cow as tonic and galactagogue.
Berberidaceae	*Berberis lycium* Royle “SBBU-42”	Koowary	Root	24	Decoction is made from its root (about ¼ kg) and is orally administered to cattle (10 days) for the treatment digestive tract infections, mouth infections, refrigerant, and healing.
Brassicaceae	*Brassica rapa* L. “SBBU-39”	Sharsham	Oil	28	Mustard oil (75 ml) and whey are mixed together and orally taken to relieve abdominal pain. This is remedy applied to cow and buffalo for the duration of 3 days.
Cactaceae	*Opuntia littoralis* (Engelm.) Cockerell “SBBU-19”	Zoqam	Whole plant	16	Powder is made from whole plant and applied topically to all domestic animals treat dermal problems, as anti-inflammatory.
Cannabaceae	*Cannabis sativa* L. “SBBU-20”	Bang	Leaves, stem	22	Leaves (½ kg) together with black tea are boiled and then mixed with wheat flour. This remedy is given to domestic animals as refrigerant and also given to the cattle suffering from genital prolapsed.
	*Celtis australis* L. “SBBU-25”	Tagha	Bark	8	Plants powder, ghee, and gurr are combined together and given to cattle twice a day (up to 3 days) for treating digestive disorders.
Chenopodiaceae	*Chenopodium album* L. “SBBU-35”	Sarmy	Whole plant	17	About ½ kg leaves are taken and boiled in mustard oil along with gurr, orally taken by goat, sheep, cow, buffalo, and donkey. This remedy is used as stomachic.
Convolvulaceae	*Convolvulus arvensis* L. “SBBU-31”	Perawaty	Whole plant	13	Fresh leaves (1 kg) and gurr are boiled in combination with black tea. This remedy is given orally (5–10 days) for milk production.
Cupressaceae	*Chamaecyparis obtusa* (Siebold & Zucc.) Endl. “SBBU-40”	Saber Dana	Fruit	9	Powder is made from its seeds and makes black tea and then utilized to heat the animals. This remedy is used for buffalos and cows for 3 days.
Cuscutaceae	*Cuscuta reflexa* Roxb. “SBBU-66”	Mecha	Whole plant	8	First make a paste of leaves and then combine with wheat flour. This remedy is then given to goats and sheep (5–10 days) as antispasmodic.
Euphorbiaceae	*Ricinus communis* L. “SBBU-68”	Aranda	Oil	15	Oil of its seed (½ cup) is administered orally for the duration of 1 week. This remedy is used as laxative and for the treatment of diarrhea.
Fabaceae	*Acacia modesta* Wall. “SBBU-27”	Palusa	Bark	11	The decoction obtained from its bark and is combined with butter which is administered orally to all type of domestic animals to treat skin problems and as blood purifier.
	*Cassia fistula* L. “SBBU-4”	Amaltas	Fruit	13	Fruit of the plant is subjected to boiling along with milk and administered orally up to 3 days to all sort of domestic cattle to relieve fever and gastric complexities.
	*Glycyrrhiza glabra* L. “SBBU-26”	Khwaga Waly	Roots	33	Root (¼ kg) is subjected to paste which is mixed with flour and oil and then is given to goat, sheep, cow, and buffalo to increase milk production and enhance the rate of fertility. The remedy is used for the duration of 5 to 7 days.
	*Lotus corniculatus* L. “SBBU-72”	FatiKhany	Stem and leaves,	19	Stem and leaves are crushed in weight of ¼ kg and orally given to cattle along with bread or dough for 7 to 10 days as sexual tonic and for urinary tract infections (UTI).
	*Trigonella foenum-graecum* L. “SBBU-11”	Malkhoozi	Seeds	9	Seeds (150 g) are crushed and given in dough to animals (5–6 days) against gastric disorders.
Fagaceae	*Quercus oblongata* D. Don “SBBU-7”	Tor batangar	Fruit	22	Kernels of this plant are given to animal for the entire cold season to keep the animals energized. Skeletal problems and internal infection are also treated by utilizing the kernels.
Juglandaceae	*Juglans regia* L. “SBBU-55”	Ghuz	Leaves	18	Fresh leaves powder (¼ kg) and gurr are thoroughly mixed. This remedy is orally taken and effective in gastric disorders.
Lamiaceae	*Ajuga integrifolia* Buch. Ham. ex D. Don “SBBU-30”	Gutee	Leaves	16	Decoction is made from the leaves and then gurr is added. This remedy is given orally to cattle for blood purification and as vormifuge. The water is applied topically to treat skin ailments.
	*Mentha spicata* L “SBBU-58”	Podina	Leaves and stem	22	Powder is made and decoction is made and then mixed with gurr and taken by animals to cure digestive problems.
	*Ocimum basilicum* L. “SBBU-9”	Kashmaly	Leaves seed	11	Plant leaves and seeds are subjected to decoction and used topically for skin problems.
	*Salvia moorcroftiana* Wall. ex Benth. “SBBU-13”	Kharghwag	Leaves	9	Decoction of its leaves is given orally daily for the treatment of digestive problems.
Malvaceae	*Gossypium arboreum* L. “SBBU-43”	Pomba	Kal	11	About ¼ kg of its powder is mixed with gurr and used for 10 to 12 days. This remedy is administered orally on daily basis as galactagogue.
	*Grewia optiva* J.R.Drumm. ex Burret “SBBU-62”	Pastawony	Whole plant	6	Dried plant powder is subjected to oil (250 ml), administered orally and topically twice a day for 5 to 7 days for wound healing process.
Meliaceae	*Melia azedarach* L. “SBBU-54”	Tora Bokana	Leaves	15	Plant leaves (200 g) are powdered and then combined with sugar. This remedy is orally given to cattle (2–3 days) against gastric disorders. Diarrhea is also treated by mixing the powder with flour cakes of maize.
Moraceae	*Ficus carica* L. “SBBU-73”	Inzar	Fruit and leaves, fruit	16	Fresh leaves (about ½ kg) are fed to animals for digestive ailments and disturbances. Fruit (¼ kg) is given two times per day for placental removal.
	*Streblus asper* Lour. “SBBU-14”	Tor tooth	Fruit	12	Fruits (2 kg) are crushed by addition of little quantity of water. This remedy is given orally to livestock for producing cooling effect.
Myrtaceae	*Eucalyptus canaldulensis* Dehnh. “SBBU-41”	Lachi	Leaves	17	Leaves decoction and gurr are thoroughly mixed and orally given to cattle (6–8 days) against digestive disorders and as appetizer.
	*Myrtus communis* L. “SBBU-23”	Mano	Leaves	18	Powder is made from it, and then, the ground leaves are mixed with whey or milk to treat digestive tract problems.
Nyctaginaceae	*Boerhavia erecta* L. “SBBU-36”	Insut	Whole plant	7	Gurr is mixed with the powder of the plant which is locally known as “Paa” and is topically applied to skin infections.
Oleaceae	*Olea europaea* L. “SBBU-46”	Zytoon	Oil	18	Oil is applied externally and emulsified to inflamed areas and broken bones. The oil is orally given for general body improvement.
Paeoniaceae	*Paeonia emodi* Royle “SBBU-69”	Mamekh	Fruit and rhizome	18	Powder is made and combined with flour which is used as tonic and for the treatment of cough in goat and cow.
Papaveraceae	*Papaver somniferum* L. “SBBU-15”	Koknar	Latex	8	Latex is dried (10 g) and boiled in black tea, which is an effective analgesic remedy. Little dried latex is combined with saliva and placed on wound for blood clotting.
Pedaliaceae	*Sesamum indicum* L. “SBBU-65”	Konzaly	Oil	8	Oil of the plant is combined with whey or milk and then given to buffalo and cow for vaginal thrush.
Pinaceae	*Cedrus deodara* (Roxb. ex D. Don) G. Don “SBBU-45”	Nanzra	Oil	19	10–15 plant oil drops are water mixed for oral administration to goats, cows, buffaloes, and sheep. This is the best remedy for the removal of bad smell of milk. The same recipe is also utilized for gastric complexities and as cooling agent. To depress the sexual ability of the animals, oil in large quantity is combined with water.
	*Pinus roxburghii* Sarg. “SBBU-53”	Nakhtar	Latex	13	Gums are water mixed and given to buffaloes, cows, and sheep against skin infections/allergies.
Platanaceae	*Platanus orientalis* L. “SBBU-10”	Chinar	Bark	14	Bark’s powder, ghee, and milk are mixed together and boiled. This remedy is orally given to cattle against gastric infections.
Poaceae	*Oryza sativa* L. “SBBU-28”	Chawal	Seed	17	Seeds (1 kg) are water boiled with yoghurt. The remedy is orally given to cattle (20–25 days) as galactagogue.
	*Triticum aestivum* L. “SBBU-51”	Ghanam	Seeds	13	Gurr is boiled with flour (¼ kg) and then given to animals to enhance rate of fertility (10–12 days).
Polygonaceae	*Rumex hastatus* D. Don “SBBU-21”	Tarukay	Whole plant	17	Root (1 kg) are taken and mixed with the powder of bark of *Quercus incana* and then boiled along with sugar and flour which is traditionally recommended for the treatment of UTI, digestive problems, and wound healing. This remedy is used for the duration of 10 to 15 days.
Primulaceae	*Primula denticulata* Sm. “SBBU-37”	Mamera	Stem	7	Stems’ decoction is topically used against eye infections.
Ranunculaceae	*Nigella sativa* L. “SBBU-34”	Kalonji	Seed	16	Decoction is made from its seeds (100 g) and given to cattle and buffaloes as sexual tonic and general body tonic.
Rhamnaceae	*Zizyphus jujuba* Mill. “SBBU-6”	Baira	Fruit and leaves,	17	Fresh leaves (100 g) decoction is orally given (2–3 days) for the removal worms. It is also used for gastric troubles and as diuretic.
	*Ziziphus oxyphylla* Edgew. “SBBU-8”	Elanai	Root	15	Decoction (fresh roots) is useful against liver infections in all types of domesticated animals.
Rosaceae	*Prunus armeniaca* L. “SBBU-2”	Khobanaiy	Gum	9	Gums are boiled with ghee and then powdered upon drying. Later on milk is added for oral administration to cattle for treating abdominal pain, for flatulence, and as appetizer.
	*Rosa moschata* Herrm. “SBBU-57”	Gulab	Flower	12	Flowers (100 g) are taken in water, and later, sugar milk are mixed into it. This remedy is orally given to livestock to produce anti-inflammation and anti-congestion effect.
Rutaceae	*Zanthoxylum armatum* DC. “SBBU-29”	Dambara	Fruit	19	Fruits powder and wheat flour are mixed and are used as antipyretic and killing mouth germs. Fruits are also considered as a good tonic for animals. This plant is also feed to the animals being suffered due to cold fever “local name Charmakh.”
Salicaceae	*Salix tetrasperma* Roxb. “SBBU-47”	Wala	Bark	16	Bark is crushed and cooked in ghee then mixing it with flour of maize which is given to all type of cattle to treat cough and internal body infections.
Simaroubaceae	*Ailanthus altissima* (Mill.) Swingle “SBBU-50”	Speena Bokana	Leaves	13	The leaves of the plant are used as galactagogue.
Solanaceae	*Nicotiana tabacum* L. “SBBU-70”	Tambacoo	Leaves	20	Decoction is made from its leaves, and then, it applied externally on animal’s body and then rubbed for external parasites. Infusion of its leaves is drenched via nostrils against leech infestation in cows.
	*Solanum virginianum* L. “SBBU-64”	Maraghuny	Roots, leaves, fruit	43	Various parts of the plant are taken (½ kg), and then, decoction is made by adding salt and yoghurt. This remedy is orally given to cattle (15–20 days) against cough, fever, and pain and for milk production.
	*Withania somnifera* (L.) Dunal “SBBU-38”	Kotilal	Leaves	38	Roots and leaves are cooked in ghee with gurr and then the resultant is given to sheep, cow, and buffalo for milk production. Also, it is used as antipyretic and sexual tonic.
Thymelaeaceae	*Daphne oleoides* Schreb. “SBBU-3”	Laighona	Leaves and flower	6	Fresh leaves and flowers are taken and are shaded dried for 15 days and subjected to powder which is then mixed with gurr and flour and given to for the expulsion of worms to cattle and buffalo.
Zingiberaceae	*Curcuma longa* L. “SBBU-1”	Korkaman	Rhizome	29	Rhizome is subjected to powder and boiled in ghee. It is orally and topically used 2 times per days (5–10 days) to relieve external and internal parasite. This is also used to treat genital infection and problems.
	*Zingiber officinale* Roscoe “SBBU-12”	Adrak	Root	11	Rhizome (80 g) is grinded and combined with gurr of 500 g. This mixture is then boiled in 2 l of milk. This remedy is traditionally considered useful for goats, cows, and sheep (taken up to 10 days) against digestive disorders, for flatulence and as appetizer.
Zygophyllaceae	*Peganum harmala* L. “SBBU-49”	Spalany	Seed, whole plant	21	Fresh leaves (about ½ kg) are burned, and then with this smoke, animals are fumigated to relieve external parasites.
	*Tribulus terrestris* L. “SBBU-56”	Azghakay	Leaves	17	Leaves (200 g) of the plant are dried and made powder of it and then mix with the powder of *Foeniculum vulgare* and administered orally to domestic animals up to 15 days to treat digestive problems.

According to a survey, out of 122 plant-derived pure compounds, 80% (94 plant species) were having the same potential as indicated in traditional medications [54]. As an example, galegine is obtained from *Galega officinalis* L. and is used in the production of metformin and other bisguanidine-type anti-diabetic drugs [55]; khellin, extracted from *V. daucoides*., led to the development of cromolyn in the form of sodium cromoglycate, which is used as a bronchodilator; and papaverine isolated from *Papaver somniferum* forms the baseline for verapamil, which is generally utilized for hypertension [55]. Survey participants did not describe the standardized dosage and recovery time like other previous ethnoveterinary documentations. The main problem highlighted in other studies is the lack of accuracy in such ethnoveterinary practices, which also push the locals towards modern allopathic drugs for livestock health maintenance [20, 56]. The main reason that veterinarian has always complained is the non-standardized dosage in traditional medicines. Though this is an accusation, one ethnomedicine does not mean that they lack efficacy but require standardization, which could benefit the traditional system by minimizing risks and toxicities. According to Kearns [57], ethnoveterinary medicines are facing a great intellectual challenge from social theory and postmodernism, and this challenge was focused while detecting variations in animal health practices, beliefs, and experiences of various social groups. Generally, it is not possible for all ethnoveterinary practices to be effective and, at the same time, they have certain weakness in terms of their efficacy as compared to modern medications [58]. Though it is convincing that most of the traditional veterinary medications have clear and sound health effects, many modern allopathic drugs are based on these medicines [59].

Certain plants in our study were used in single form for more than one disease. For example, *Cedrus deodara* (Roxb. ex D. Don) G. Don is used in a condition, in which milk obtained from the cattle gives bad smell, then the oil is given orally to the cattle. It is also used as a cooling agent and in treating digestive problems. In large quantity, the oil have the potential to depress the sexual power of male animals [49]. Monteiro et al. [60] also reported similar findings from Pakistan and Brazil, respectively, where they described multiple uses of a single medicinal plant. Utilization of certain plant species for multiple diseases is a widespread practice in ethnoveterinary medications. In contrast, some ethnoveterinary remedies (polyherbal formulations) are being made by combing two or more plants and additives such as whey, ghee, and sugar. This addition is generally followed in remedies to counteract the astringent taste, dilute, and reduce the relative potency of the remedy [61].

### Ethnoveterinary disease category

In the study area, a total of 32 plants were reported for gastrointestinal problems with maximum use reports of 433 (Table 3), which is regarded as the most common disease category in domestic animals being represented by abdominal pain, diarrhea, and digestive problems. These health issues can be easily detected by the respondents and may explain the fact that why the gastric problem category is high in ours as well as in others studies. Different ailments were categorized into 10 groups such as dermatological, gastrointestinal, galactagogue, reproductive, respiratory disorders, tonic, wound healing fever, and miscellaneous. Those medical conditions, which were not fully described by the interviewees, were placed into the miscellaneous category. These include eye problems, weakness, and abnormal conditions related to various organ systems of animal bodies. Highest Fic values were recorded for dermatological problems (0.97) followed by reproductive ailments (0.93) and gastric disorders (0.92) (Table 3). Fic value is an indicator of showing the consent of the local people on a specific plant species and efficacy of a certain taxa [62]. Sharma et al. [63] declared that when Fic becomes 1, it means that the local population is exchanging their view, ideas, and information about traditional medications, while on the other side, if the Fic value is 0, then it is vice versa. Fic value in the current study was recorded in between 0.85 and 0.97 for various livestock ailments (Table 3). These findings indicate the highest consent among the local people on traditional herbal therapies. Previous research studies conducted in other areas also agreed to high consent of local people on traditional animal therapies. For instance, the reported Fic values for dermatological problems were 0.93, 0.93, and 0.82 [26, 64, 65]; for reproductive disorders, 1.00 and 0.89 [65, 66]; for gastric problems, 0.90, 0.70, 0.92, 0.95, and 0.94 [26, 64–66]; for galactagogue, 0.83 and 0.50 [6, 65]; and for wound healing, 0.40 and 0.45 [6, 67]. Heinrich et al. [68] has submitted the idea that high Fic values can be used as a tool to target the plants for the isolation of biologically active components. In our study, most livestock’s ailments were mentioned to be seasonal and epidemic due to change in fodder. Furthermore, the concept of hot and cold food is also famous in order to prevent animals from diseases. The local residents change the relative fodder in different seasons in order to minimize the chances of various health problems in cattle. As an example, the seeds of the *Nigella sativa* L. and kernels of *Q. oblongata* are given to the cattle to energize them during the cold season. Similarly, the fruits of the *Streblus asper* Lour. produce cooling effects and considered to be a better remedy during hot summer season. In the same manner, local communities tend to give the infusion of *Cannabis sativa* L to their livestock in the summer season. Quinlan [69] and Raziq et al. [9] has also mentioned the concept of hot and cold food in traditional veterinary medications.

**Table 3:** Category wise informant consensus factor (Fic) (Bajaur Agency)

Medical categories	Number of species	Citations	Fic
Tonic	5	38	0.89
Dermatological	4	120	0.97
Gastrointestinal	32	433	0.92
Galactagogue	8	86	0.91
Reproductive	11	155	0.93
Miscellaneous	22	201	0.89
Respiratory disorders	4	37	0.91
Fever	5	41	0.90
Wound healing	7	41	0.85

### Pharmacological evidences

Drugs derived from plants or their extracts have certain therapeutic properties. To replace antibiotics by suitable therapeutic agents, plants can play an important role in combating with bacterial pathogens. There are several essential oils, which can be used as alternate of antibiotics. These oils can be easily isolated, having low toxicity on mammalian cells, and can be easily degraded in soil and water [70]. In this section, we will analyze the pharmacological evidences of the most utilized studied medicinal plant species in order to check their therapeutic efficacy.

In *F. vulgare*, phenols, phenolic glycosides, and volatile aroma compounds such as transanethole, estragole, and fenchone are reportedly the key phytoconstituents and responsible for its antioxidant activity. *F. vulgare* is pharmacologically validated (in vitro and in vivo) in demonstrating activities such as antibacterial, antifungal, antioxidant, antithrombotic, and hepatoprotective [71]. By investigation, it was found that the leaf extracts of *S. virginianum* is more active against *Candida albicans*, *Salmonella typhi*, *Staphylococcus aureus*, and nematodes [72, 73]. For various extracts obtained in alcohol and water, it was found that *W. somnifera* has antibacterial potential, antihypercholesterolemic activities as well as diuretic potential [72, 74].

It has been reported that alcoholic and aqueous extracts of *C. longa* have shown antibacterial activity [74] while its ethanol, petroleum, water, and chloroform extracts are effective against certain strains of viruses, bacteria, and fungi and also have shown anti-inflammatory effects [75]. Researchers have claimed that plant-derived medicines used in traditional systems across the globe can be used as an indicator to consider them more effective than modern drugs [6]. Livestock keepers are using several plant-derived remedies for various acute as well as chronic disorders of cattle. Plant-derived medicines have been used by physicians for hundreds of years in traditional systems, and most of the world population rely on these products for health care systems [76]. There are several thousand plants across the globe being utilized for various therapeutic purposes both animals and humans [49]. Out of these medicinal plants, very low proportion has been investigated and proved scientifically for their Indigenous uses [77]. The essential oils in medicinal plants are having strong antimicrobial potential. As an example, essential oils of cinnamon, thyme, and oregano are therapeutically effective [78].

Antibiotic resistance is an emerging global concern related to veterinary and human medications [79]. Hence, it is necessary to search for new compounds to combat antibiotic resistant bacteria. Improper therapeutic utilization of antimicrobial medicines in fishery, poultry, agriculture, and animal farming facilitates the emergence and production of drug resistant strains. Additionally, poor prevention and control of unhygienic practices contribute in resistance emergence. The World Health Organization, Food and Agriculture Organization, and World Organization for Animal Health are stressing to promote best practices to avoid the emergence and spread of antibacterial resistance. Continuous attempts are in progress to promote the moderate use of antibiotics in human as well as in animals to tackle the problem of antimicrobial drug resistance [80].

In general, plants should be used as an alternative to synthetic drugs and investigated for their therapeutic efficacy. Certain plants in our study including *Boerhavia erecta* L., *Celtis australis* L., *Chamaecyparis obtusa* (Siebold & Zucc.) Endl., *Eryngium biehersteinianum* (M. Bieb.), *Gossypium arboreum* L., *H. candicans* Wall. ex DC., *Narcissus tazetta* L., *Opuntia littoralis* (Engelm.) Cockerell, and *S. asper* need comprehensive phytochemical, pharmacological, and toxicological investigations.

### Current study, one health concept, and changing environment

Current study reports that there are several ailments being treated with medicinal plants by the Indigenous populations. Most prevalent disease categories were dermatological, reproductive, and gastric problems. The dominance of these diseases not only poses threats to the domestic animals but also increases the chances of zoonoses. Local population uses various animal products; hence, there are maximum chances of the migration of infectious diseases from these animals to humans. Linkage of the ethnoveterinary studies with the researches of other disciplines may form an interdisciplinary approach to combat several types of health issues in both animals and plants. This approach mainly led to the concept of one health, which contributes towards understanding the complexities in health problems of living beings [81]. A recent surge in emerging infectious diseases and their putative associated costs to society have reignited interest in the drive of disease emergence. A number of pathogens have emerged in the last 20 years, including the severe acute respiratory syndrome virus, Hendra virus, and Nipah virus. However, there is a growing concern about the H_5_N_1_ influenza virus, which fuelled much of the recent debate around emerging infectious diseases (EIDs) [82]. One of the benefits that accrued from the attention on EIDs has been an increased recognition across a range of disciplines that the health of animals (including humans) and the health of the broader ecosystem are inextricably linked, which certainly given momentum to One Health movement. One Health is not all about EIDs, but it also covers important issues of food security and food safety [83]. There is a strong consensus that the climate is changing now and that human activities are the primary cause [84]. However, it is clear that climate change will alter the distribution and incidence of a wide range of diseases either directly or indirectly (e.g., diseases with a development stage outside the host) [85, 86]. The pathways by which climate change can affect host pathogen vector interactions have recently been well described by Gallana et al et al. [86].

One Health Initiative Task Force (OHITF) [87] defines one health as “the promotion, improvement, and defense for the health and well being of all species by enhancing cooperation and collaboration between physicians, veterinarians, and other scientific health professionals and by promoting strengths in leadership and management to achieve these goals”. The one health approach plays a significant role in the prevention and control of zoonoses. Approximately 75% of new emerging human infectious diseases are defined as zoonotic [79, 88]. Of the 1461 infectious diseases, approximately 60% are caused by multi-host pathogens, characterized by their movement across various species [89]. This gives significant credence to the importance of examining health effects across species, in order to fully understand the public health and economic impact of such diseases and to help implement treatment and preventive programs.

The application of one health approach has been recognized as a critical need by international organizations as well as the preferred approach to address global health issues. It is also noted that knowledge in veterinary medicine and animal nutrition and husbandry could provide insights into human nutrition and growth.

### Biodiversity concerns

It is a widespread phenomenon that natural resources including plants are always prone to threats in their natural habitat due to rapid human intervention and destructions of natural resources. The collection process of medicinal plants for ethnic practices and other anthropogenic practices is not only destructing the Indigenous flora but also posing a threat to the traditional knowledge. UNESCO has emphasized on the documentation and preservation of traditional knowledge in South Asia generally and Pakistan and India particularly. However, efforts are going on but they are not sufficient for the conservation of traditional knowledge persistent since several centuries, which can lead to valuable discoveries in modern healthcare system. The local perception of Indigenous communities regarding the threats being faced to the ecological resources especially the medicinal plants was examined in the current study. The lack of awareness has been observed as a major threat to the conservation of plant resources. It was also observed that different factors including time of collection, processing, storage, and herbal preparations are important and necessary steps to be considered for both economic returns and conservation. Mainly, the local healers are involved in the collection of medicinal plants. A study in the Swat region of Pakistan has shown that higher economic outcomes can be obtained from proper harvesting of wild medicinal plants as compared to the standard cash crop [90]. Other studies are supporting our results by showing an enormous potential in improving the harvesting, storage, use, preparation, and marketing of the herbal product as a source of income [91]. In the remote areas of the study region, local inhabitants obtained significant economic advantages from forest products. Similar advantages have been reported for other mountainous communities in the northern parts of Pakistan [26].

There are certain other threats to the medicinal plant resources of the study area, which include deforestation, heavy grazing pressure, uncontrolled collection of fodder, and other non-timber forest products by the local people and traders. Several studies have reported a decrease in the number of medicinal plants due to over exploitation and environmental degradation [92, 93]. It is therefore a dire need to manage and design the overall grazing system to encourage the sustainable regeneration and protection of medicinal plants. Keeping the observation and findings of the current investigation, proper management steps should be taken with the active participation from the Indigenous communities to conserve this precious flora. It is also important to aware the local people about the market value and sustainable harvesting of medicinal plants. Rapid modernization and urbanization is not only a threat for plant species’ degradation but also a threat for the associated folk knowledge. That is why that the disappearance of folk knowledge has been declared more in danger than the natural resources themselves [94]. Therefore, we present a strong recommendation that ethnobotany as a subject should be included into the curriculum to help students in recognizing the endangered and medicinally important species of their respective regions. In addition, incentives may be given to farmers for the cultivation of medicinal plants on marginal lands and home gardens.

## Conclusions

Indigenous communities at Bajaur Agency are dependent on medicinal plants for ethnoveterinary practices. Knowledge about the traditional medicinal system is restricted to the herders, farmers, and elder community members. The younger generation is unaware of this traditional treasure and takes no interest due to modernization. Hence, this study is an attempt towards the preservation of traditional ethnoveterinary knowledge from being extinct. There are several medicinal plants, which are being used in traditional herbal system of veterinary disorders. Some of the important are *V. daucoides*, *F. vulgare*, *S. virginianum*, *W. somnifera*, *G. glabra*, and *C. longa*. New ethnoveterinary uses used at the study area were found for *H. candicans* and *G. glabra*. Apiaceae is utmost plant family being in use for various livestock ailments. Thorough phytochemical and pharmacological investigations are required by isolating the active compounds and testing the in vitro or in vivo efficacy of the abovementioned plants against the targeted veterinary diseases. Furthermore, critical toxicological investigations are also required to ensure the safe and secure use of documented ethnomedicines. In order to share and further maintain this knowledge, it is direly needed to aware the rural population about the significance of traditional ethnoveterinary knowledge and to motivate them on the conservation of natural flora*.*

## Abbreviations

EIDs: Emerging infectious diseases
FATA: Federally administrated tribal areas
Fic: Informant consensus factor
GDP: Gross domestic product
OHITF: One Health Initiative Task Force
UNESCO: United Nations Educational, Scientific and Cultural Organization
URs: Use reports
